# Cue predictability does not modulate bottom-up attentional capture

**DOI:** 10.1098/rsos.180524

**Published:** 2018-10-31

**Authors:** Erik L. Meijs, Felix H. Klaassen, Levan Bokeria, Simon van Gaal, Floris P. de Lange

**Affiliations:** 1 Radboud University Medical Center, Donders Institute for Brain, Cognition and Behaviour, 6500 HB Nijmegen, The Netherlands; 2 Donders Institute for Brain, Cognition and Behaviour, Radboud University, 6500 HB Nijmegen, The Netherlands; 3 Department of Psychology, University of Amsterdam, 1001 NK Amsterdam, The Netherlands; 4 Amsterdam Brain and Cognition (ABC), University of Amsterdam, 1001 NK Amsterdam, The Netherlands

**Keywords:** bottom-up attention, attentional capture, expectation, predictability

## Abstract

Attention can be involuntarily captured by physically salient stimuli, a phenomenon known as bottom-up attention. Typically, these salient stimuli occur unpredictably in time and space. Therefore, in a series of three behavioural experiments, we investigated the extent to which such bottom-up attentional capture is a function of one's prior expectations. In the context of an exogenous cueing task, we systematically manipulated participants' spatial (Experiment 1) or temporal (Experiments 2 and 3) expectations about an uninformative cue and examined the amount of attentional capture by the cue. We anticipated larger attentional capture for unexpected compared to expected cues. However, while we observed attentional capture, we did not find any evidence for a modulation of attentional capture by prior expectation. This suggests that bottom-up attentional capture does not appear modulated by the degree to which the cue is expected or surprising.

## 1. Introduction

When performing tasks in our everyday lives, we constantly have to battle potential distraction by task-irrelevant inputs. Even when we want to stay focused on the task at hand, it can be difficult to ignore other, often more salient, stimuli that capture our attention. Historically, there has been considerable debate on whether attentional capture is purely stimulus-driven [1,2], or also depends on top-down goals [3]. More recently, it has been suggested that recent trial history [4] and associations with reward [5,6] may also modulate attentional capture. This implies that the presence and amount of attentional capture may be a complex function of both stimulus and internal variables [7].

Stimulus expectation is another factor that may modulate bottom-up capture. One of the studies providing evidence for this was carried out by Folk & Remington [8]. In a spatial cueing paradigm, they manipulated the frequency of salient but uninformative (i.e. not predictive of the target) cues. Their results indicated that these cues captured attention only when they were unlikely, regardless of the top-down task set participants were using (but see [9] for an alternative interpretation). In another study, the proportion of distractors was systematically varied over blocks [10]. The distractors interfered more with target processing when they were presented in a block with fewer distractors, suggesting they captured attention more when they were more surprising. Similarly, it has been observed that novel stimuli are most potent in capturing attention [11] and also most robustly modulate the neural response in a macaque's V1 [12]. Taken together, these studies support the hypothesis that attentional capture by task-irrelevant stimuli may be modulated by perceptual expectations, and most notably by the violation of these expectations. This can be interpreted as evidence that surprising stimuli are more salient and therefore more attention-grabbing [13,14]. Additional evidence supporting this idea comes from studies on mismatch detection, in which it has been shown that unexpected deviant stimuli lead to larger mismatch responses in the EEG-signal and seem to subjectively ‘pop out’ [15–17].

Besides influencing the amount of attentional capture by distracting stimuli, there is evidence that prior information about these cues can help participants to voluntarily diminish distraction [18,19]. For example, it has been shown that attentional capture by unlikely distractors can be attenuated when the search task promotes suppressing features similar to those of the distractors' stimuli [9]. Another study showed that it is easier to ignore regular sequences than irregular sequences [20]. Whether reducing the amount of distraction is caused by the inhibition of attentional capture, or by rapid disengagement at a later stage is still debated [4,21]. An electrophysiological study by Kiss and colleagues suggested that bottom-up capture can be inhibited, but that this only happens when task demands (i.e. timing) require it [22].

One interpretation of the empirical evidence above is that surprising stimuli are more salient and therefore more attention-grabbing [13,14]. Predictive coding theories have suggested that processing unexpected events requires more resources [23,24]. One may conceptualize bottom-up attention as a way of redistributing resources, for example, towards processing unexpected events. This is in line with findings that bottom-up attention increases contrast sensitivity [25]. Nevertheless, many models based on predictive coding have actually suggested that regularity and predictability may attract attention. The idea behind this is that predictable inputs are more strongly weighted because they are more reliable. A number of studies have provided support for this idea [26], suggesting that regularities automatically attract attention (but see e.g. [27]).

It thus seems that the link between expectation and bottom-up attention is still far from clear [24,28]. Attention may be either drawn to surprising stimuli, or to regularly occurring ones. Therefore, we performed a series of three experiments using an exogenous cueing task [29], in which we explicitly looked at this relationship by manipulating participants' expectations about an otherwise uninformative (i.e. unrelated to target) cue stimulus. Specifically, we investigated to what extent prior expectations about the cue modulated bottom-up attentional capture. Based on the evidence listed earlier, we anticipated that unexpected cue stimuli will attract more attention and therefore result in larger cue-target validity effects (i.e. performance difference between validly and invalidly cued trials), whereas expected cue stimuli are followed by strongly reduced or even absent validity effects. To preview, in contrast to this hypothesis, we observed attentional capture in all experiments, but no direct modulation by prior knowledge about the cue stimulus in any experiment.

## 2. Experiment 1: Do spatial expectations affect bottom-up attention?

### 2.1. Methods

#### 2.1.1. Participants

We tested 120 participants in Experiment 1. This number was based on power analysis for a between-subjects design with 40 participants per group, with 80% power to detect medium-sized effects. All participants had normal or corrected-to-normal vision. We excluded two participants whose task performance was markedly (more than 3 s.d.) worse than that of other subjects. As a result, we included 118 participants (87 females, age 22.7 ± 5.0 years).

#### 2.1.2. Materials

Stimuli were presented using the Psychophysics Toolbox [30] within MATLAB (MathWorks, Natick, MA, USA), generated by a Dell T3500 Workstation and displayed on a 24″ BENQ LED monitor (1920 × 1080 pixels; 60 Hz; screen size 53.1 cm × 29.9 cm). All presented stimuli were ‘black’ (RGB: [0 0 0]; ±0.3 cd m^−2^) on a grey (RGB: [150 150 150]; ±103.9 cd m^−2^) background. A chinrest was used to control the distance participants were seated from the monitor (±57 cm). Participants responded by means of two button boxes.

#### 2.1.3. Procedure and stimuli

**Figure 1.**
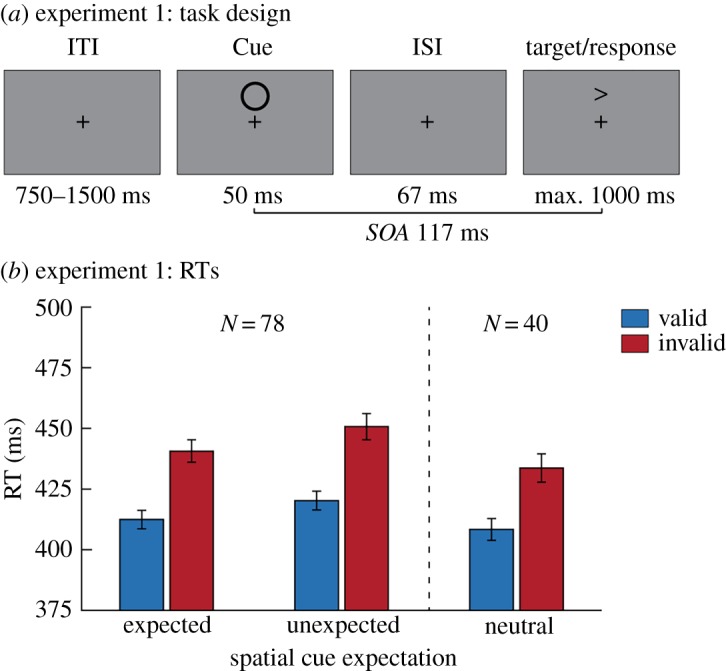
Task design and behavioural results of Experiment 1. (*a*) Trial structure of the exogenous cueing task used in Experiment 1. In every trial a cue (circular annulus) was presented for 50 ms, either above or below fixation. After an inter-stimulus interval (ISI) of 67 ms (stimulus-onset asynchrony (SOA) 117 ms), a target (arrow) was presented in either the same (valid trials) or opposite (invalid trials) location. We manipulated spatial cue expectation by varying the likelihood the cue would appear in either location. In one group of participants, the cue appeared equally often above and below fixation. In the two other groups, the cue was more likely (80%) to appear in one of the locations. Target location was counterbalanced and unrelated to the cue location. The participants' task was to report the direction the arrow was pointing in. (*b*) Reaction time (RT) results for Experiment 1. Only trials in which the correct answer was given were used for the analysis. On the left, we show results for participants that expected the cue either above or below fixation (*N* = 78), meaning that it was sometimes presented in the expected location and sometimes in the unexpected location. Participants were faster on valid than on invalid trials, regardless of their spatial expectations about the cue. For reference, we present the results for participants in the neutral group (*N* = 40) on the right. Error bars represent s.e.m.

Participants performed an adjusted version of the exogenous cueing task ([29], figure 1*a*). First, a cue (2° circular outline) was presented for 50 ms either 5° above or below fixation. After the cue, a target was presented centred on either same (valid trials) or the opposite (invalid trials) screen location. Cue location and target location were unrelated, meaning that both target locations were equally likely throughout the experiment, regardless of where the cue was presented. Targets were small (0.48° wide and 0.60° high) arrows pointing either leftward or rightward. The participants' task was to report the direction the arrow was pointing in (leftward or rightward) by pressing a button with either their left or right index finger, while maintaining fixation throughout the experiment.

In Experiment 1 we manipulated the likelihood that the cue would appear either above or below fixation. In two groups of participants, the cue was most likely to appear, respectively, above or below fixation. Consequently, participants in these two groups (*N* = 78) encountered both trials where the cue was in the expected location (80%) as well as trials where it was in the unexpected location (20%). In a third group of participants (*N* = 40), both cue locations were equally likely, resulting in those participants experiencing only neutral trials.

The stimulus-onset asynchrony (SOA) between the cue and target was set to 117 ms. The target remained onscreen until a response was given or until 1000 ms after target onset had passed. Trials were separated by a variable inter-trial interval of 750–1500 ms. Participants responded to the arrows by pressing a button on a button box with their left or right index finger, respectively, for leftward and rightward pointing arrows.

In total, the experiment lasted approximately 1 h. Before starting with the main experimental task, participants received onscreen instructions and performed one practice block of 80 trials. Participants were told that the cue was irrelevant (i.e. non-predictive of the target) and could be ignored. During the practice block, participants received onscreen feedback (response correct or incorrect) on a trial-by-trial basis. Subsequently, participants performed 800 trials of the main task divided into 10 blocks. After each block, participants received feedback about their task performance (overall percentage correct and number of late responses) and subsequently there was a short 20 s break.

#### 2.1.4. Behavioural analysis

Trials where the participants' reaction time exceeded 3 s.d. from the participants' mean or was below 200 ms were discarded. Furthermore, trials on which no (relevant) response was given within 1 s from target onset were excluded from analyses. The remainder of the trials (98.3%) were exported to JASP [31] in order to perform statistical analyses. We analysed the data using a combination of both frequentist statistics and their Bayesian equivalents [32,33].

For the statistical analyses of Experiment 1, we performed a 2 × 2 repeated-measures ANOVA with the factors Validity (valid, invalid) and Expectation (expected, unexpected) for the reaction times. This analysis only considered participants for whom the cue was more likely to appear in one of the locations (*N* = 78). Participants in the neutral condition were included as a reference group, to be able to interpret possible performance differences as either gains or losses in performance with respect to an expectation-neutral context. In two independent samples *t*-tests, we compared the validity effect size between the neutral condition and the expected or unexpected condition in the test group participants. In addition to frequentist analyses, we also computed Bayes Factors for all relevant comparisons. As we were specifically interested in the interaction between expectations and the bottom-up validity effect, the Bayes Factor (BF) for a model with only the main effects was compared to the BF for a model with the main effects and the interaction. The ratio of the BF values then quantifies the evidence for including the interaction term in the model, and hence can be interpreted as evidence for or against the existence of an interaction between the two experimental factors. BF ratios will converge either to infinity when a model including the interaction explains the data better, or to zero when it explains the data worse than a model with only main effects. If the ratio is close to one, this indicates that both models are equally likely and that there is not enough evidence for either conclusion. We use the conventions from Jeffreys [34] to interpret the evidence in our Bayesian analyses.

### 2.2. Results and discussion

We examined whether expectations about the spatial location of a cue modulate the ensuing attentional capture by this cue. In figure 1*b* we plot the reaction time results for Experiment 1 for trials on which participants gave the correct answer. Higher reaction times on invalid than valid trials indicate there was attentional capture by the cue (RT difference = 29.31 ms, *F*_1,77_ = 216.53, *p* < 0.001, *η*^2^=0.738). Importantly, however, this validity effect was not modulated by the spatial expectation participants had about the cue (*F*_1,77_ = 0.753, *p* = 0.388). The evidence against the existence of this modulation is moderate (BF_01_ = 4.40). While spatial expectations did not modulate attentional capture, there was an overall reaction time benefit for trials when the cue was in the expected location compared to the unexpected location (RT difference = 8.87 ms, *F*_1,77_ = 84.56, *p* < 0.001, *η*^2^ = 0.523). Further *post hoc* analyses showed that the validity effect in the neutral control group was not significantly different from that on either expected (*t*_116_ = −0.96, *p* = 0.338) or unexpected (*t*_116_ = −1.15, *p* = 0.252) trials for participants who had expectations about cues. Accuracy was close to ceiling level (98.21 ± 1.21% correct) and is further reported and analysed in the supplementary material available online.

Together, these results suggest that our manipulation of spatial expectations modulated overall behavioural speed and accuracy but did not result in a modulation of bottom-up attention capture. While this suggests that expectations do not interact with bottom-up attention, there are possible alternative explanations. Most notably, because targets and cues are often presented in the same location, and with only a short (117 ms) and predictable time delay between the two, suppressing processing at the cue location would possibly hamper target processing. Moreover, because the cue was temporally predictive of the target (fixed SOA), its presentation was informative about target onset and hence attending the cue at all times may have been useful for target perception. These considerations inspired Experiment 2, in which we examined whether cues still elicit bottom-up attention when they are temporally predictable but no longer predictive of when targets occur.

## 3. Experiment 2: The effect of temporal expectations on bottom-up attention

### 3.1. Methods

#### 3.1.1. Participants

We tested 67 participants in Experiment 2. Compared to Experiment 1, fewer participants were tested because a within-subjects design was used. All participants had normal or corrected-to-normal vision. We excluded four participants from Experiment 2 because button presses were not recorded properly. As a result, we included 63 participants (50 females, age 23.0 ± 3.5 years) in the final analysis.

#### 3.1.2. Materials

We used the same materials as in Experiment 1, with the only exception being that participants now used the computer keyboard (DELL KB522) to respond.

#### 3.1.3. Procedure

**Figure 2.**
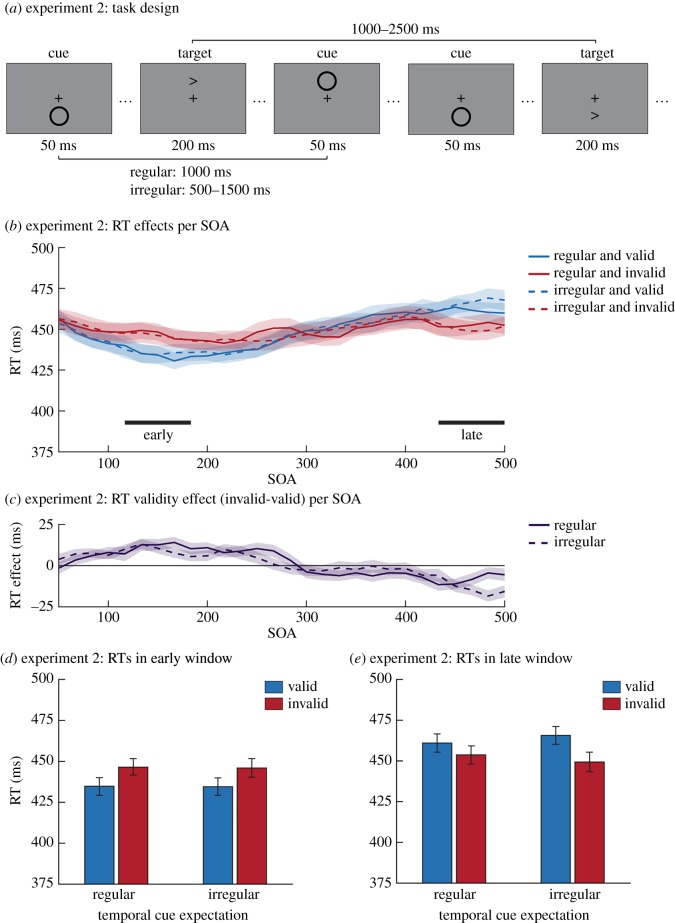
Task design and behavioural results of Experiment 2. (*a*) Example stimulus sequence for Experiment 2. The task consisted of continuous blocks (duration 300 s) during which cues and targets were presented. In some blocks, cue presentation was regular and hence expectations about cue onset were strong. In other blocks, cue presentation was less expectable. Again, the participants’ task was to report the direction the target arrow was pointing in. (*b*) The reaction time results and validity effect (invalid-valid) (temporally smoothed) for SOA bins between 50 and 500 ms are shown for each of the relevant conditions. Below the graph, the time periods that were isolated as windows (around 150 and 467 ms) for further analyses are marked. (*c*) The validity effect (invalid–valid) results (temporally smoothed) after regularly and irregularly presented cues for SOA bins between 50 and 500 ms. The average reaction times per condition from the windows in (*b*) are presented in (*d*) for the early window and (*e*) for the late window. In both windows, there was a significant validity effect that was not affected by temporal cue expectations. Error bars represent s.e.m.

Instead of manipulating spatial expectations about the cue, in Experiment 2 we manipulated temporal expectations. To remove standard temporal links between cues and targets, the task consisted of continuous blocks (duration 5 min) during which cues and targets were presented (figure 2*a*). Temporal expectations were manipulated by varying the regularity of cue presentation onset between blocks. In the regular (expected condition) blocks, the cue would be presented every second (1 Hz presentation rate). In the irregular blocks (unexpected condition) cues were presented quasi-randomly every 0.5–1.5 s, with a uniform distribution over all possible intervals between two consecutive cues. This results in the participant having less (precise) information about the cue onset and the cue-target SOA. Targets lasted 200 ms and were presented quasi-randomly every 1.0–2.5 s. Importantly, the onsets of cues and targets were determined completely independently of each other. Because the SOA between two targets was longer than between cues, not every cue was followed by a target. Participants used the ‘z’ and ‘/’ (slash) buttons on the keyboard to respond to leftward and rightward pointing arrow targets, respectively.

Participants completed eight blocks of the task, switching between regular and irregular conditions after every two blocks. The initial condition was counterbalanced over participants. After every block, there was a 20 s break. If a condition switch occurred, this was explicitly mentioned onscreen at the end of the break. Participants were instructed that the cue was irrelevant to their task and could be ignored. Participants did not receive any feedback during the task. Together with the instructions and the practice block, the experiment lasted approximately 1 h.

#### 3.1.4. Behavioural analysis

During the experiment, the task was presented to participants in continuous streams, without a clearly discernible trial structure. For analytical purposes, trials were defined *post hoc* by isolating at all target presentations (on average 1371 trials per participant). Data preprocessing was similar to Experiment 1 and resulted in the inclusion of 96.0% of trials. Subsequently, for each target presentation, we identified the temporal distance between the target and the closest preceding cue stimulus. This generated a total of 90 bins, spanning a cue-target SOA between 0 and 1500 ms, in 17 ms steps (SOAs above 1000 ms were infrequent, less than 10% of trials). The first button press after the target presentation was recorded as the participants' response. If no response was made within a 1000 ms interval following the target, the trial was classified as a ‘miss’ trial.

Because the number of trials per condition was low (due to a large amount of possible SOA bins; on average 20.6 trials per SOA bin in Experiment 2 and 21.0 trials per SOA bin in Experiment 3), we smoothed the data in the temporal domain by applying a sliding window over all SOA bins of interest (SOAs below 500 ms). In this procedure, for every relevant combination of conditions, the data we ascribe to an SOA are computed as a weighted average from that SOA (SOA_0_) and the two SOAs immediately preceding and following it (0.1 × SOA_−2_ + 0.2 × SOA_−1_ + 0.4 × SOA_0_ + 0.2 × SOA_+1_ + 0.1 × SOA_+2_). Trials with an SOA between 0 and 50 ms were excluded from the analyses because in those trials cue and target presentation overlapped in time.

Based on the smoothed data, we computed the overall validity effect for each SOA. We then identified two SOAs of interest based on these data: (i) an early maximally facilitatory validity effect (SOA with a maximally positive overall validity effect); and (ii) a late maximally inhibitory validity effect (SOA with a maximally negative overall validity effect). We then averaged the (non-smoothed) data around these SOAs of interest, using the selected SOA and the two SOAs preceding or following it, to create an early and a late window of interest. Subsequently, we tested for an interaction between expectation and the bottom-up validity effect by performing a 2 × 2 repeated-measures ANOVA with the factors Validity (valid, invalid) and Expectation (expected, unexpected) in each of these windows. In addition, as in Experiment 1 the Bayesian equivalent of the ANOVA was performed. Note that because cue onsets and target onsets were determined independently, not every participant had observations for every condition at every SOA. In case data was missing in one of the conditions of an analysis, we excluded the respective participant from that analysis.

### 3.2. Results and discussion

We temporally smoothed the data over the different SOA bins (figure 2*b*). Subsequently, we identified an early and a late window where bottom-up attention effects were most prominent (see also Behavioural analysis). The windows can be interpreted as resulting from initial bottom-up attentional capture (early window), followed by inhibition of return (late window; [35]). In these windows, we tested for modulations by temporal expectations (figure 2*c,d*).

As in Experiment 1, we observed strong evidence for bottom-up attentional capture as indexed by the validity effect (early window: RT difference = 10.54 ms, *F*_1,60_ = 24.77, *p* < 0.001, *η*^2^ = 0.292; late window: RT difference = −11.18 ms, *F*_1,59_ = 15.44, *p* < 0.001, *η*^2^ = 0.207). Note that this effect is to be anticipated, because we chose our windows based on the validity effect size. Again, temporal expectations did not modulate bottom-up attentional capture, as shown by the absence of an influence of cue onset regularity on the validity effect (early window: *F*_1,60_ = 0.02, *p* = 0.883; late window: *F*_1,59_ = 2.85, *p* = 0.097). The evidence against such modulations was moderate for the early window (BF_01_ = 5.14). For the late window, likely related to the inhibition of return, there was only anecdotal evidence (BF_01_ = 2.27), suggesting the study's power was not sufficient to make any strong claims about effects in this time window. Unlike Experiment 1, there was no significant main effect of expectations on reaction time (early window: *F*_1,60_ = 0.45, *p* = 0.504; late window: *F*_1,59_ = 0.07, *p* = 0.793). Overall participants' task performance was close to the ceiling (94.00 ± 3.30%).

Cues in the regular condition were temporally predictable but spatially unpredictable, i.e. they could equally likely occur above or below fixation. It is possible that cues need to be both temporally and spatially predictable for attentional capture to be reduced. This is what we set out to test in Experiment 3, in which we replicated Experiment 2 while keeping the cue location constant.

## 4. Experiment 3: The role of temporal expectations in bottom-up attention for spatially predictable stimuli

### 4.1. Methods

#### 4.1.1. Participants

We tested 61 participants in Experiment 3. All participants had normal or corrected-to-normal vision. Two participants were excluded because their performance was markedly (more than 3 s.d.) worse than that of other subjects. In addition, one participant was excluded because of a failure to respond in more than 20% of the trials. In the end, 58 participants were included in the analyses (42 females, age 22.7 ± 3.5 years).

#### 4.1.2. Materials

The materials used were identical to those in Experiment 2.

#### 4.1.3. Procedure

**Figure 3.**
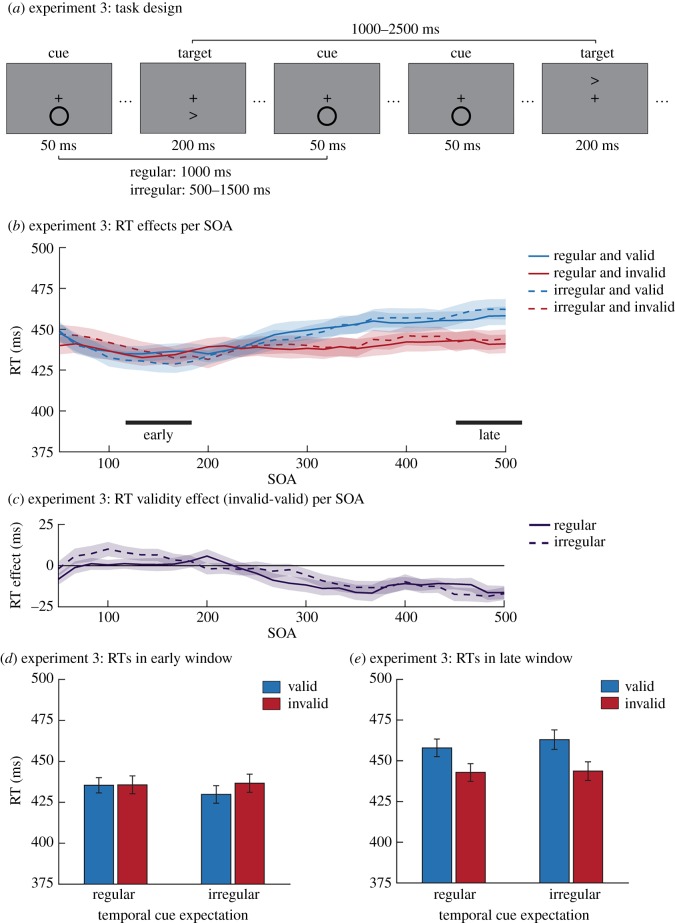
Task design and behavioural results of Experiment 3. (*a*) Example stimulus sequence for Experiment 3. The task that was used was highly similar to that in Experiment 2. The main difference was that for each participant the cue was now consistently presented in one location, making it completely spatially expected. (*b*) The reaction time results (temporally smoothed) for SOA bins between 50 and 500 ms are shown for each of the relevant conditions. Below the graph, the time periods that were isolated as windows (around 150 and 483 ms) for further analyses are marked. In the early window (*c*) there was no significant bottom-up attentional capture, regardless of temporal expectations. The late window (*d*) did show a significant effect, but this inhibition of return effect was not modulated by temporal cue expectations. Error bars represent s.e.m.

The design of Experiment 3 was largely similar to that of Experiment 2. The most notable difference was that now for each participant the cue location was kept constant throughout the task (figure 3*a*). This allowed us to investigate the effects of temporal expectations in a context where the distractor is spatially fully predictable. The cue location was counterbalanced across participants. To limit possible carry-over effects, we only switched between the regular and irregular conditions halfway through the experiment. In addition, a short practice block was included at the start of each of the conditions to get participants used to the change in task structure.

#### 4.1.4. Behavioural analysis

As in Experiment 2, trials were defined around each target (on average 1365 trials per participant). Data preprocessing steps (97.0% of trials included in analyses) and statistical analyses were identical to those in Experiment 2.

### 4.2. Results and discussion

In this final experiment, we created a condition in which cues were both spatially and temporally fully predictable. For each participant, the cue now always appeared in a single location and thus was always spatially expected. After defining the windows of interest (similar analysis pipeline as Experiment 2; figure 3*b*), we again tested whether temporal predictability modulated the bottom-up attention effects (figure 3*c,d*). As in the previous experiments, no such modulations were found (early window: *F*_1,55_ = 1.40, *p* = 0.241; late window: *F*_1,57_ = 0.96, *p* = 0.331). The evidence against these interactions was moderate for both windows (early window: BF_01_ = 3.07; late window BF_01_ = 3.91). Similar to Experiment 2, there were no main effects of temporal cue expectations on reaction times (early window: *F*_1,55_ = 0.09, *p* = 0.763; late window: F_1,57_ = 1.21, *p* = 0.276). Surprisingly, while there was significant inhibition of return (late window validity effect: RT difference = −17.11 ms, *F*_1,57_ = 30.38, *p* < 0.001), there was no overall attentional capture in this experiment (early window validity effect: *F*_1,55_ = 0.09, *p* = 0.763) and the evidence against the existence of such an effect was moderately strong (BF_01_ = 3.37). As in the previous experiments, participants’ overall task performance was close to the ceiling (95.50 ± 2.72%).

## 5. Comparisons between experiments

**Figure 4.**
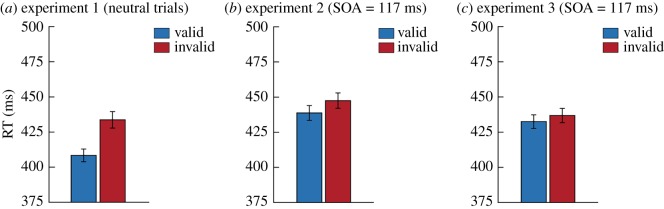
Comparing the overall validity effects between experiments. For each of the experiments, we display the average reaction times on valid and invalid trials for the selections of trials that were used to compare validity effects between the experiments. (*a*) Only the neutral condition of Experiment 1 (control group) was used. The SOA in this experiment was fixed at 117 ms. For experiments 2 (*b*) and 3 (*c*) we only used the data we obtained for the SOA of 117 ms after smoothing the SOA time courses. Error bars represent s.e.m.

Because the experiments differed markedly in the expectations participants had about cues and the context in which those cues were presented, we compared the validity effect sizes between experiments. Therefore, we performed two *post-hoc* analyses in which we directly compared reaction time validity effects between experiments. To ensure maximal comparability, for Experiments 2 and 3, we take the validity effect at an SOA of 117 ms (after smoothing), which is the SOA that was used in Experiment 1. First, we compared Experiment 1 (only neutral trials; figure 4*a*) and Experiment 2 (figure 4*b*). In terms of spatial expectations both are comparable (cue 50% in each location), but the experiments differ strongly in the temporal context in which stimuli are presented. Most notably, in Experiment 1 the SOA was fixed at 117 ms, while in Experiment 2 it was variable and unpredictable. Therefore, in Experiment 1, the cue was a good temporal predictor of target onset. A comparison of the validity effects by means of an independent samples *t*-test shows a significantly smaller validity effect in Experiment 2 than in Experiment 1 (*t*_101_ = 4.47, *p* < 0.001, *d* = 0.904). This is possibly explained by that fact that in Experiment 2 there was (i) less information about the onset of the target and (ii) a lower likelihood (not after every cue) a target would appear.

Second, the absence of an initial bottom-up capture effect in Experiment 3 (figure 4*c*) could be potentially explained by the perfect predictability of cue locations in the experiment. Because in contrast to Experiment 3 cue location was unpredictable in Experiment 2, the comparison between those experiments can be used to test this hypothesis. An independent samples *t*-test showed that there was no significant difference between the validity effects (*t*_119_ = 1.182, *p* = 0.240) of both experiments. Therefore, we did not find evidence that the bottom-up validity effect is modulated by spatial expectations about the cue. This conceptually replicates our findings of Experiment 1. However, it should be noted that there is only anecdotal evidence (BF_01_ = 2.75) that the validity effects of Experiments 2 and 3 were of equal size and these results should thus be interpreted with caution.

## 6. Discussion

In a series of three behavioural experiments, we investigated whether bottom-up attentional capture is modulated by stimulus expectations. We did not observe empirical support for this hypothesis. On the contrary, in all experiments, we found moderately strong evidence that the bottom-up validity effects were of comparable size when cue stimuli were expected, compared to when they were not (or less) expected.

The fact that participants were overall faster on expected compared to unexpected trials in Experiment 1 suggests that participants did form prior expectations, which had a sizeable influence on behaviour. Based on studies showing that stimuli embedded in regular streams are better detected [26], we anticipated a similar main effect of expectation for the temporal paradigm in Experiments 2 and 3. Contrary to our predictions, we did not observe this effect, even though regular and irregular blocks were visibly different and every switch between conditions was explicitly marked in the participants' instructions. Other studies have used similar paradigms with streams of stimulation to investigate effects of temporal expectations and did find effects of temporal regularity on subsequent behavioural performance [20,36]. Nevertheless, temporal expectations about cues do not appear to influence the processing of subsequently presented target stimuli in situations where targets are salient and uncoupled from the cues.

Assuming that our manipulations of expectations actually instantiated priors in our subjects, the absence of any significant interaction between expectations and bottom-up attention in our experiments is surprising, because it contradicts the hypothesis that unexpected or surprising events capture attention (more) [11,13,14]. Interestingly, two recent studies observed that distractor predictability can modulate the amount of attentional capture in a visual search task where target and distractor are presented concurrently [37,38]. The most notable difference in task design between their study and ours is that in their paradigm distractors and targets were presented simultaneously at different locations of the screen, resulting in direct attentional competition between the stimuli. This competition can be biased by predictability. By contrast, in our studies, we examine the consequences of presenting a salient cue stimulus in isolation on subsequent visual processing at that location. We find that the attention-grabbing properties of such a cue is not modulated by predictability. In line with our findings, a recent study by Southwell and colleagues has also suggested that regular and random streams are equally salient [27]. Moreover, our results are in line with the stimulus-driven account of bottom-up attention [2,39], in which it is assumed that the initial capture of attention is automatic and independent of top-down factors. However, it must be noted that even within the stimulus-driven account there would have been room for expectations to suppress the effects of distracting inputs (i.e. the uninformative cues) at later processing stages [4,21].

One conceivable alternative explanation for the absence of effects of expectation on attentional capture is that the tasks we used were too simple. Each participant's performance was close to ceiling (greater than 90%) in all experiments. As a result, ignoring or suppressing the uninformative cues may not have been required in order to perform well. Indeed, recent studies [22,40] showed that attentional capture was only suppressed if task requirements were such that capture would interfere with target processing. Moreover, a study in macaques showed that modulations of V1 attention responses were larger for tasks that were more difficult [12].

Furthermore, a recent study has suggested that only fully spatially predictable distractors may be suppressed at an early processing stage [41]. This hints at the possibility that our manipulation of expectations, especially in Experiment 1, was not potent enough to influence bottom-up capture. It is possible that with a stronger manipulation of expectations (i.e. making cues even more likely in one condition and less likely in the other) we would have observed a modulation of capture. Still, this cannot fully explain the absence of an effect in Experiment 1: even in Experiment 3 when cue stimuli were perfectly predictable in terms of timing, location and visual characteristics, no significant modulation of the validity effect was found.

It is noteworthy that ignoring or suppressing the uninformative cues in our tasks may generally not have been a useful strategy. It is conceivable that participants did not inhibit the cue location at any point in a trial, because a target would often (50%) be presented in the same location with only a short time delay. As a consequence, ignoring one location systematically would be detrimental to target detection. Moreover, the fixed SOA in Experiment 1 resulted in cue onset being perfectly predictive of target onset time. Hence, paying attention to an informative cue, instead of ignoring it, was actually a viable strategy. Consequently, in all experiments, the tasks we used may have had factors that made participants attend cues (instead of ignoring them). In addition, cues and targets were both defined as abrupt onset stimuli, meaning cue features to some extent overlapped with the searched-for target feature [3]. It is conceivable that this overlap caused attention to be captured regardless of the experimental condition. As a result, potential effects may have been obscured because the task set did not optimally support suppression of uninformative cues [9].

We observed a significant difference in the amount of attentional capture between Experiment 1 and Experiment 2. While it is difficult to directly compare both experiments because they differed in several dimensions, a likely candidate explanation for this difference is the fact that in Experiment 2 there was more (temporal) uncertainty about the onset of the targets, as well as an overall lower likelihood of target appearance. It is conceivable that participants deploy a different strategy in Experiment 2 compared to Experiment 1, in which they focus more on the targets and less on the cues (because those are less/not informative), which in turn leads to the cue having less influence on subsequent target processing. Future studies are required to test this idea.

In conclusion, we did not find evidence for modulations of bottom-up capture by spatial or temporal expectations about the cue. We therefore conclude that, at least in the exogenous cueing tasks we used, bottom-up attentional capture does not seem to be altered by prior knowledge about the location or time point of the distracting inputs. This calls into question perceptual surprise as an explanation for bottom-up attention. Future research may use more difficult tasks in which the relationship between targets and distractors can be more carefully controlled. In addition, we believe electrophysiological studies could possibly disentangle the effects of expectation and attention and precisely point at their interactions with high temporal resolution.

## Supplementary Material

Supplementary analyses: Percentage correct results

## Data accessibility

The behavioural data and analysis scripts used in this article are available at the following web address: http://hdl.handle.net/11633/di.dccn.DSC_3018012.14_675. Prior to accessing and downloading the shared data, users must create an account. It is possible to use an institutional account or a social ID from Google+, Facebook, Twitter, LinkedIn or Microsoft. After authentication, users must accept the Data Use Agreement (DUA), after which they are automatically authorized to download the shared data. The DUA specifies whether there are any restrictions on how the data may be used. Radboud University and the Donders Institute for Brain, Cognition and Behaviour will keep these shared data available for at least 10 years.
