# Supplementary material for: Cue predictability does not modulate bottom-up attentional capture
Source: R Soc Open Sci. 2018 Oct 31;5(10):180524. doi: 10.1098/rsos.180524 (PMC6227932; doi:10.1098/rsos.180524)
Supplement: Supplementary analyses: Percentage correct results [file rsos180524supp1.pdf]

Supplementary analyses: Percentage correct results

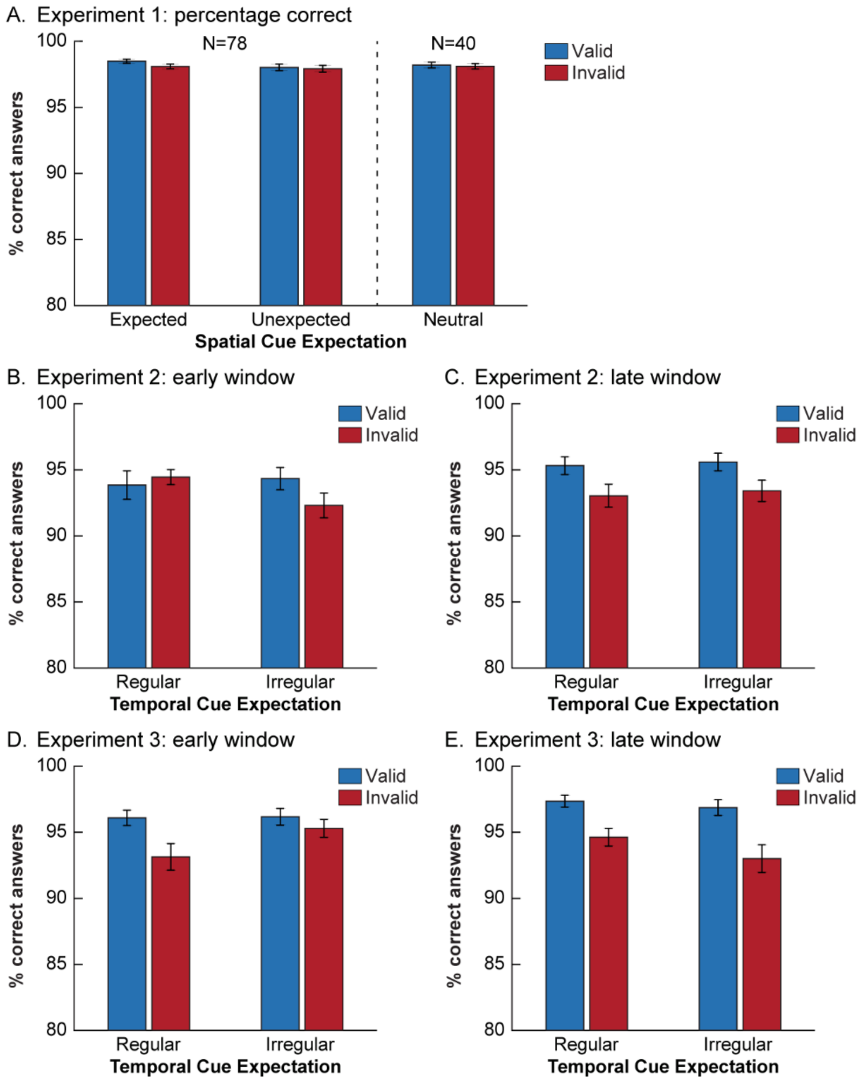

Supplementary Figure 1. Percentage correct results. (A) Percentage correct responses in Experiment 1. On the left we show results for participants that expected the cue either above or below fixation (N=78), meaning that it was sometimes presented in the expected location and sometimes in the unexpected location. For reference, we present the results for participants in the neutral group (N=40) on the right. Below, we present the percentage correct results for Experiment 2 **(BC)** and Experiment 3 **(DE)**, separately for the early and late time windows. Error bars represent SEM.

Experiment 1

Participants' performance was higher on expected than unexpected trials (Supplementary Figure 1A; $F_{1,77}$=5.02, p=0.028, η^2=0.061). However, there was no validity effect ($F_{1,77}$=2.10, p=0.152), regardless of spatial expectations about the cue ($F_{1,77}$=0.56, p=0.458).

Experiment 2

We used the same time-windows as for the reaction time analyses. Percentage correct was not significantly affected by temporal expectations (early window: $F_{1,60}$=1.22, p=0.275; late window: $F_{1,61}$=0.28, p=0.596). Cue validity did impact on task performance, but only in the late time window (early window: $F_{1,60}$=1.10, p=0.299; late window: $F_{1,61}$=12.93, p<0.001, η^2=0.175). Moreover, the validity effect size was not significantly modulated by temporal expectations about the cue in either of the windows (early window: $F_{1,60}$=3.29, p=0.075; late window: $F_{1,61}$=0.03, p=0.873).

Experiment 3

We again used the same time-windows for the accuracy analyses as for the reaction time analyses. Expectations had a significant effect on participants' performance in the early window ($F_{1,55}$=4.25, p=0.044, η^2=0.072) but not in the late time window ($F_{1,57}$=2.31, p=0.134). In both windows, the validity of the cue significantly affected performance (early window: $F_{1,55}$=5.36, p=0.024, η^2=0.089; late window: $F_{1,57}$=23.32, p<0.001, η^2=0.290). Importantly though, in neither of the windows this validity effect was modulated by temporal expectations (early window: $F_{1,55}$=2.19, p=0.145; late window: $F_{1,57}$=0.80, p=0.374).
